# Comparison of Three Mathematical Models of the Pharmacokinetics of Heparin to Guide the Use of Protamine in a Large Simulated Cohort of Patients

**DOI:** 10.1177/10892532251332464

**Published:** 2025-04-10

**Authors:** Stanislaw Vander Zwaag, Imre Kukel, Asen Petrov, Jens Fassl

**Affiliations:** 1 Department of Cardiac Anaesthesiology, Heart Centre Dresden, 39074 TUD Dresden University of Technology, Dresden, Germany; 2 Department of Cardiac Surgery, Heart Centre Dresden, 39074 TUD Dresden University of Technology, Dresden, Germany

**Keywords:** heparin, protamine, pharmacokinetics, cardiopulmonary bypass, cardiac anaesthesia

## Abstract

**Background:** In cardiac surgery, protamine is used to reverse the effects of heparin after separation from cardiopulmonary bypass (CPB). Excess of protamine has been demonstrated to cause platelet dysfunction and coagulopathy. A protamin-to-heparin ratio of less than 1:1 is endorsed by the European guidelines. Pharmacokinetic models of heparin decay have been proposed to allow for individualised dosing rather than fixed ratios. The objective of this study is to compare three such models in a large cohort of simulated patients. **Methods:** The doses of protamine were calculated using the models proposed by Meesters et al., Miles et al., and in the PRODOSE trial. We employed data from the VitalDB database to calculate the doses of heparin and randomly generated time intervals in-between. We tested two scenarios: with an initial dose and heparin added to the priming solution, and where additional heparin was administered over the course of the CPB. **Results:** We simulated 1166 cases with a mean interval between heparin and protamine administration of 90 ± 22 minutes in the first and 140 ± 28 minutes in the second scenario. The PRODOSE formula produced the lowest protamine-to-heparin ratios, followed by Meesters’ formula in the first scenario (0.68:1 vs 0.72:1, *P* < .001) and the Miles’ formula in the second scenario (0.55:1 vs 0.62:1, *P* < .001). **Conclusion:** The doses calculated with pharmacokinetic models exhibited discrepancies of up to 13.6%. While confirmation of complete reversal with alternative methods is necessary, the models facilitate a more individualised dose selection than the fixed ratios proposed in the literature.

## Introduction

The administration of heparin in large doses to prevent blood clotting within the cardiopulmonary bypass is a defining aspect of cardiac anaesthesiology. These doses frequently exceed 500 units per kilogram in total. Following the separation from the cardiopulmonary bypass (CPB), the excessive heparin in the system must be reversed to avoid ongoing bleeding. Traditionally, a protamine-to-total-heparin ratio of 1:1 has been employed. However, the administration of protamine carries itself inherent risks regarding the coagulation status.

While protamine inhibits heparin by causing dissociation of the heparin-antithrombin complex, it also has the effect of reducing thrombin generation, reducing platelet count and function, and enhancing fibrinolysis.^1-3^ Accordingly, the surplus of protamine relative to the circulating heparin levels may exert a deleterious impact on the coagulation process. The European Association of Cardiothoracic Surgery (EACTS) and European Association of Cardiothoracic Anaesthesia (EACTA) Guidelines on patient blood management for adult cardiac surgery have determined that a ratio of less than 1:1 should be employed in the majority of cases, citing literature for ratios in the range of 0.6–0.8:1.^4,5^ Nevertheless, the evidence to support other fixed ratios remains inconclusive.^6-8^

The pharmacokinetics of heparin are complex and highly dependent on the injected dose. Heparin is eliminated from the bloodstream via two principal mechanisms: rapid renal elimination and uptake by the reticuloendothelial system. The latter occurs mainly in the Kupffer cells of the liver and the sinus endothelial cells of the spleen.^9^ The kinetics of heparin elimination are non-linear. In a small study comprising four participants in each group, the half-life of a dose of 200 units per kilogram was approximately 96 minutes, while a dose of 400 units resulted in an approximate half-life of 152 minutes.^10^ A search, conducted on PubMed and Google Scholar in December 2024 using the keywords ‘heparin half-life', did not identify any studies of higher doses conducted in humans.

One approach to individualise the dosing of protamine is the development of pharmacokinetic models and their utilisation to calculate the amount of heparin decayed over the course of the cardiopumonary bypass. Examples of such models include the one-compartment model proposed by Miles and colleagues, as well as the two-compartment models proposed by Meesters et al. and in the *Optimal protamine dosing after cardiopulmonary bypass* (PRODOSE) trial.^11-13^ The models differ in terms of the number of theoretical compartments in which heparin is distributed, as well as in terms of the distribution and elimination rates, as evidenced by the half-lives that have been utilised. In their trials, the authors of the aforementioned models employed a 1:1 protamine-to-heparin ratio based on the estimated amount of heparin present in the system at the time of protamine administration. An overview of the analysed models is presented in Table 1.

**Table 1.** Overview of the Compared Pharmacokinetic Models.

Model	Number of Compartments	t_1/2_ for Fast Compartment	t_1/2_ for Slow Compartment
Meesters et al., 2016^11^	Two	10 minutes	250 minutes
Miles et al., 2017^12^	One	N.A.	$26+0.323\left(\frac{Heparin\;dose}{\;Ideal\;body\;weight\;}\right)$
PRODOSE 2021^13^	Two	10 minutes	$26+0.323\left(\frac{Heparin\;dose}{\;Ideal\;body\;weight\;}\right)$

Abbreviation: t_1/2_, Half-Life.

The objective of this study is to compare the protamine doses calculated with the three presented models in a large sample size, comprising a number of simulated cases with different doses of protamine and different durations of extracorporeal circulation.

## Methods

Three models of pharmacokinetics of heparin were compared in our study: the one-compartment model described by Miles et al.,^12^ and the two-compartment models described by Meesters et al.^11^ and in the PRODOSE trial.^13^

The high-fidelity open-access VitalDB database of real-world patients published on PhysioNet was employed to provide demographic data for a large cohort of surgical patients.^14-16^ This database comprises anonymised records of patients who underwent surgical procedures at Seoul National University Hospital between August 2016 and June 2017. The acquisition and subsequent publication of data for further research were approved by the Institutional Review Board of the aforementioned hospital. We selected patients aged 18 years or above undergoing all types of surgeries who required postoperative intensive care unit stay from the database and included them in the further analysis. The ideal body weight for each patient was calculated using the Devine formula.^17^

The objective of the study was to compare the hypothetical doses calculated with the three models in two different scenarios. In these simulated scenarios, the typical dosages employed in our institution were utilised. The first scenario (Scenario 1) involved the administration of 500 units of heparin per kilogram of total body weight prior to cardiopulmonary bypass, with an additional 5000 units incorporated into the priming solution of the CPB, and no further heparin administered throughout the course of extracorporeal circulation. The second scenario (Scenario 2) represented a more complex case, with a longer duration of CPB. It entailed the same initial dose and priming solution, but with an additional 5000 units administered as a bolus during the CPB.

To reflect a variety of surgical situations, the duration of each interval between heparin and protamine doses was generated randomly using formulas within an Excel spreadsheet (Microsoft Excel, Microsoft, Redmond, WA, USA). The generated values were designed to adhere to a normal distribution around a specified mean and with a specified standard deviation. These values were selected based on typical time ranges experienced at our centre. The formulas were employed to randomly generate the duration of the intervals between the administration of the initial heparin dose and the commencement of CPB, and between CPB initiation and the administration of protamine. In the second scenario, the time between CPB initiation and the third heparin dose was additionally generated. Table 2 presents the means and standard deviations employed in the generation of the intervals.

**Table 2.** Means and Standard Deviations Employed to Generate the Data for the Duration of the Intervals Used in the Calculations.

	Scenario 1		Scenario 2		
	t_IC_	t_CR_	t_IC_	t_C2_	t_2R_
Mean (min)	20	70	20	70	70
SD (min)	8	20	8	20	20

Abbreviations: t_IC_, time between initial dose of heparin and initiation of the cardiopulmonary bypass; t_CR_, time between the initiation of the cardiopulmonary bypass and the reversal of heparin; t_C2_, time between the initiation of the cardiopulmonary bypass and the administration of the second dose of heparin; t_2R_, time between the administration of the second dose of heparin and the reversal of heparin; SD, standard deviation.

For each simulated case and scenario, the dose of protamine for heparin reversal was calculated in accordance with the formulas presented in each paper. The calculated doses were rounded up to the nearest ten milligrams. Furthermore, the ratios of calculated doses to initial and total heparin doses were calculated.

Exemplary cases of each scenario were presented on concentration-time plots, thereby facilitating a visual comparison of the decay curves. Furthermore, we plotted a modified formula described by Meesters et al., utilising the t_1/2_ of 150 minutes instead of 250 minutes stated in the original formula. The t_1/2_ in the PRODOSE formula is dependent on the initial dose of heparin; therefore, we plotted three curves with doses of 300 units/kg, 400 units/kg and 500 units/kg, respectively.

The primary outcome of the study is the total dose of protamine for heparin reversal, calculated using the cited formulas. Secondary outcome of the study are the ratios of protamine to initial heparin and of protamine to total heparin administered during the case.

### Statistical Methods

The statistical analysis of the data was conducted utilising the JASP 0.19 software (JASP Team, Amsterdam, The Netherlands). Descriptive statistics are employed to present the data. A paired Student's t-test is employed to compare data that is normally distributed. Data that is not normally distributed is compared with a Wilcoxon signed-rank test. Normally distributed data are presented as mean ± standard deviation, whereas non-normally distributed data are presented as median [interquartile range]. A *P*-value of less than 0.05 is assumed to be statistically significant.

## Results

### Participants and Descriptive Data

Out of a total of 6388 patients recorded in the VitalDB database being considered, 1166 patients were selected for inclusion in the study. The demographic data, along with the calculated ideal body weight and the generated data regarding the cardiopulmonary bypass, are summarised in Table 3. In Scenario 1 the mean total duration between the initial heparin bolus and the administration of protamine was 90 ± 22 minutes (range 19–166 min), in Scenario 2160 ± 28 minutes (range 31–237 min).

**Table 3.** Demographic Data of the Selected Patients From the VitalDB Database Along With the Generated Data Regarding the Cardiopulmonary Bypass.

	Mean ± SD, Median [IQR], or Count (%)
Age, years	64 [56–73]
Sex, male	751 (64%)
Height, cm	164 [157.5–169.2]
Weight, kg	59.7 [52.5–67.1]
BMI, kg • m^−2^	22.6 [20.4–24.9]
IBW, kg	60 [50.6–65.0]
Initial dose of heparin, units	30 000 [27 000–34,000]
**Scenario 1**	
t_IC,_ min	20.1 ± 7.7
t_CR,_ min	69.7 ± 20.3
Total dose of heparin, units	35 000 [32 000–39,000]
**Scenario 2**	
t_IC,_ min	20.0 ± 8.1
t_C2,_ min	70.6 ± 20.5
t_2R,_ min	69.0 ± 20.0
Total dose of heparin, units	40 500 [36 000–43,000]

Abbreviations: BMI, body mass index; IBW, ideal body weight; t_IC_, time between the initial dose of heparin and the initiation of the cardiopulmonary bypass; t_CR_, time between the initiation of the cardiopulmonary bypass and the reversal of heparin; t_C2_, time between the initiation of the cardiopulmonary bypass and the administration of the second dose of heparin; t_2R_, time between the administration of the second dose of heparin and the reversal of heparin.

### Main Results and Other Analyses

**Figure 1.**
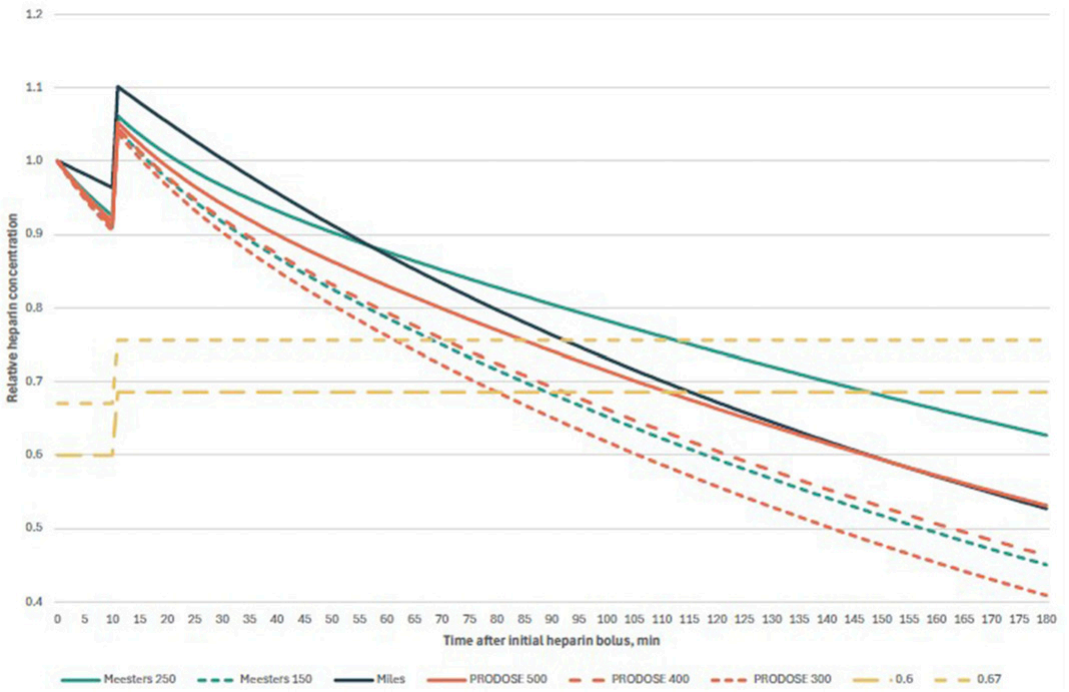
Comparison of exemplary heparin decay curves in Scenario 1. After initial bolus of heparin (in this case, 35 000 units), cardiopulmonary bypass was commenced after 10 minutes, with 5000 units heparin in the priming solution. Abbreviations: Meesters 250, original formula of Meesters et al. with t1/2250 minutes; Meesters 150, modified formula of Meesters et al. with _t1/2_ 150 minutes; Miles, formula of Miles et al.; PRODOSE 500, 400, and 300, formula from PRODOSE trial with 500, 400, and 300 units of heparin per kg, respectively; 0.67 and 0.6, two-thirds and six-tenths of the total heparin dose, respectively.].

**Figure 2.**
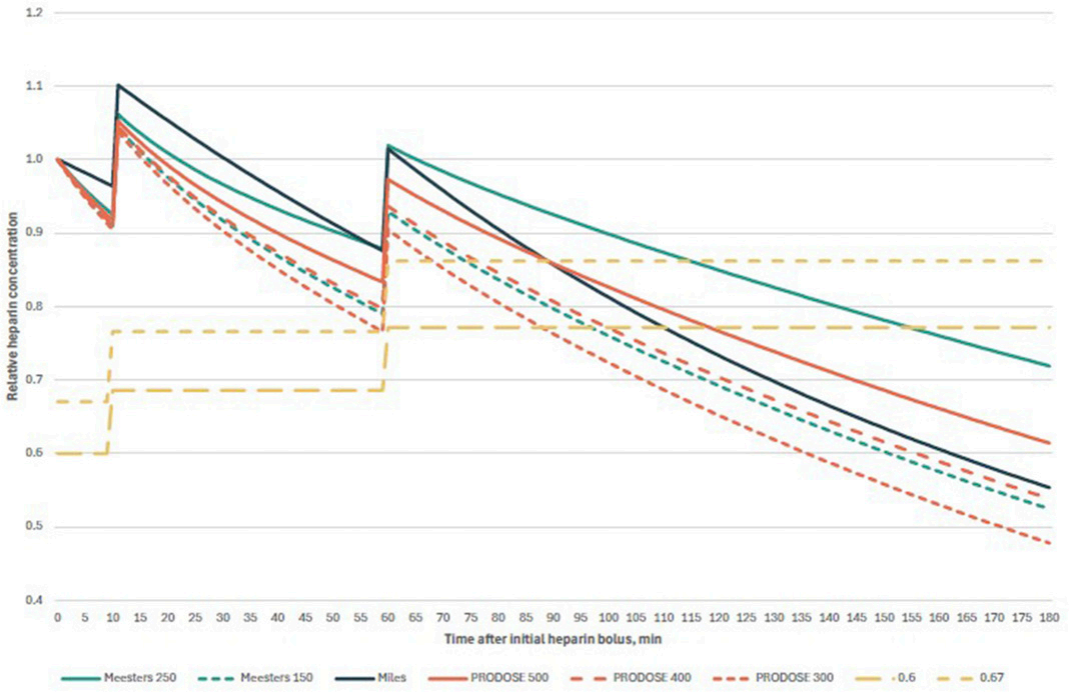
Comparison of exemplary heparin decay curves in Scenario 2. After initial bolus of heparin (in this case, 35 000 units), cardiopulmonary bypass was commenced after 10 minutes, with 5000 units of heparin in the priming solution; 50 minutes thereafter another 5000 units of heparin were administered; Abbreviations: Meesters 250, original formula of Meesters et al. with t_1/2_ 250 minutes; Meesters 150, modified formula of Meesters et al. with t1/2150 minutes; Miles, formula of Miles et al.; PRODOSE 500, 400, and 300, formula from PRODOSE trial with 500, 400, and 300 units of heparin per kg, respectively; 0.67 and 0.6, two-thirds and six-tenths of the total heparin dose, respectively.].

The calculated protamine doses exhibited statistically significant differences between the models proposed by Meesters et al. and Miles et al. (*P* < .001), Meesters et al. and PRODOSE (*P* < .001), and Miles et al. and PRODOSE (*P* < .001). In both scenarios under examination, the doses calculated with the PRODOSE model were the lowest, followed by the model proposed by Meesters and colleagues in Scenario 1 and that put forth by Miles and colleagues in Scenario 2. The comprehensive results of the statistical analyses are presented in Table 4. The decay curves for illustrative cases for both scenarios are provided in Figures 1 and 2.

**Table 4.** Comprehensive Results of the Statistical Analysis. All Pairwise Comparisons Were Statistically Significant With *P* < .001.

	Meesters et al.^11^	Miles et al.^12^	PRODOSE^13^
**Scenario 1**			
Total protamine dose, mg	260 [230–290]	270 [230–300]	240 [210–270]
Protamine : Initial heparin ratio	0.85:1 [0.81–0.88]	0.88:1 [0.830–0.930]	0.79:1 [0.75–0.84]
Protamine : Total heparin ratio	0.72:1 [0.69–0.75]	0.75:1 [0.71–0.80]	0.68:1 [0.64–0.72]
**Scenario 2**			
Total protamine dose, mg	250 [220–270]	240 [210–260]	220 [190–240]
Protamine:initial heparin ratio	0.83:1 [0.78–0.88]	0.82:1 [0.76–0.88]	0.74:1 [0.69–0.79]
Protamine:total heparin ratio	0.62 :1 [0.59–0.65]	0.61:1 [0.57–0.65]	0.55:1 [0.51–0.58]

## Discussion

The findings of this study of two modelled scenarios of on-pump surgery on a sample of 1166 patients demonstrate that the protamine doses calculated using the PRODOSE formula are consistently the lowest among all the compared models. In Scenario 1, with a mean total duration between the initial heparin bolus and the administration of protamine of 90 minutes (range 19–166 min), the ratio of protamine to total heparin ranged from 0.46:1 to 0.97:1 across all models. In contrast, in Scenario 2, the mean duration was 160 minutes (range 31–277 min), and the ratio ranged from 0.41:1 to 0.83:1. The ratios were observed to be the highest in cases with the shortest duration.

Despite the disparate assumptions underlying the various study models, the resulting calculations yielded comparable outcomes. In Scenario 1, the discrepancy between the lowest (240 mg in the PRODOSE model) and the highest (270 mg in the Miles’ model) mean calculated protamine dose was 12.5%. In Scenario 2, with 220 mg in the PRODOSE model and 250 mg in the Meesters model, the discrepancy was 13.6%. The issue of whether disparities of such magnitude possess any clinical significance remains unresolved and necessitates investigation in real-life studies.

The half-lives for slow compartment calculated in the Miles’ model and PRODOSE trial (155.2 minutes for 400 units per kilogram ideal body weight and 90.6 minutes for 200 units per kilogram ideal body weight) are in close agreement with the half-times reported by Olsson and Stig in their study.^10,12,13^ Conversely, Meesters et al. postulated that the impact of non-reversed heparin is more deleterious than that of an excess of protamine. To circumvent the potential underdosing of protamine in cases of intraoperative hypothermia, he employed a prolonged half-time of 250 minutes.^11^ Nevertheless, it should be noted that the study also involved cardiopulmonary bypass procedures conducted in patients under normothermic or mild hypothermic conditions, with a core temperature over 34°C. It is important to acknowledge that the model has not been validated for application in cases of moderate or deep hypothermia. In fact, the population of the PRODOSE study had a lower mean core temperature of 33.8 ± 1.5°C. As illustrated in Figures 1 and 2, the decay curve generated by substituting the half-life value in Meesters’ model with 150 minutes exhibits a high degree of similarity to the curve calculated using the PRODOSE model, which involved the administration of 400 units of heparin per kilogram of ideal body weight.

The examined models permitted the calculation of the protamine dose on an individualised basis, thereby achieving the ratios recommended by the current guidelines, which are less than 1:1.^4^ Nevertheless, it is essential to take into account the characteristics of the study population in the original trials, as well as the exclusion criteria employed by their authors. The study conducted by Meesters and colleagues excluded patients who had undergone emergency surgery, were underweight or severely overweight, had haematological disorders, or required renal replacement therapy or preoperative heparin.^11^ The Miles’ model was not evaluated in patients with preoperative laboratory coagulation abnormalities, surgeries with hypothermic circulatory arrest, surgeries on the aorta other than the aortic root, or implantations of ventricular assist devices.^12^ In the PRODOSE study, patients were additionally excluded if they were under the age of 18, had a total body weight exceeding 120 kg, had received adenosine diphosphate receptor antagonists within seven days of surgery, had received an unfractionated heparin infusion or therapeutic low molecular weight heparin within 24 hours before surgery, or were undergoing solid organ transplantation.^13^

Hypothermia leads to a deceleration in the rate of heparin elimination.^18,19^ Cohen et al. demonstrated that at 25°C, there is no notable decline in heparin concentration.^18^ Consequently, patients who have undergone surgery in hypothermia may require a greater dose of protamine than would be predicted by the models. Further studies are needed to explore the pharmacokinetics of heparin during hypothermia.

It should also be noted that the calculations are based on a heparin initial dose of 500 units per kilogram. Lower doses result in a shorter half-life in the Miles and PRODOSE models, which would further increase the differences between these models and the Meesters’ model.

In a recent randomised control trial comparing a fixed dose of 250 mg protamine with a 1:1 ratio, researchers found no differences in post-protamine ACT or chest tube output in the first 24 hours postoperatively.^20^ The fixed dose chosen by the study authors is close to the median doses calculated using the models analysed, although it is still higher than the calculated dose in some patients.

The efficacy of heparin reversal must be monitored through the use of activated clotting time (ACT) measurements or viscoelastic methods, as some patients across all trials demonstrated the necessity for additional protamine following the initial reversal procedure.

### Limitations

The present study aimed to compare protamine dosages administered under three proposed heparin pharmacokinetic models in a large model sample of patients in two clinical scenarios. It should be noted, however, that it did not encompass a comprehensive range of clinical scenarios. Nevertheless, the protamine doses calculated with the PRODOSE model will consistently be the lowest of the three models analysed, due to the presence of a fast compartment (in comparison to the Miles’ model) or a shorter half-time (in comparison to the Meesters’ model). Furthermore, the mathematical models offer only an approximation of the heparin concentration at the time of protamine administration. To confirm the efficacy of the calculated doses, it is essential to employ clinical assessment of oozing and recognised methods to assess completeness of heparin reversal.

### Future Perspectives

It is hypothesised that the pharmacokinetic models of heparin, when employed to guide protamine administration, may facilitate individualisation of dosage in accordance with the prevailing European guidelines. Further real-life studies are required to substantiate the safety of this approach, particularly with regard to the incidence of cases where the calculated dose of protamine results in underdosing. In the event of adapting one of the models within an institution, a before-after study may be conducted to assess the real-life impact.

## Conclusion

In the simulated cases, the doses of protamine calculated with pharmacokinetic models exhibited discrepancies of up to 13.6%. All the models that were studied yielded a ratio of protamine to heparin less than 1:1. The discrepancies between the models were attributable to the employment of single vs two-compartment models, as well as the utilisation of constant vs dose-dependent half-lives of heparin. While confirmation of complete reversal with alternative methods is still necessary, the models facilitate a more individualised dose selection than the fixed 1:1, 0.8:1, 0.6:1 or other ratios proposed in the literature.

## Supplemental Material

Supplemental Material - Comparison of Three Mathematical Models of the Pharmacokinetics of Heparin to Guide the Use of Protamine in a Large Simulated Cohort of Patients Supplemental Material for Comparison of Three Mathematical Models of the Pharmacokinetics of Heparin to Guide the Use of Protamine in a Large Simulated Cohort of Patients by Stanislaw Vander Zwaag, Imre Kukel, Asen Petrov, and Jens Fassl

## Data Availability

All raw data are made available as Supporting materials.

## Appendix

### List of Abbreviations

CPB: Cardiopulmonary bypass
VitalDB: Vital Signs DataBase
PRODOSE: Optimal protamine dosing after cardiopulmonary bypass trial
ACT: Activated clotting time
